# Report of the Committee on Contagious and Infectious Diseases*Read before the United States Veterinary Medical Association.

**Published:** 1889-10

**Authors:** A. W. Clement

**Affiliations:** Chairman


### ART. XXII.—REPORT OF THE COMMITTEE ON CONTAGIOUS AND INFECTIOUS DISEASES.*

By A. W. CLEMENT, V.S., *Chairman.*

The use of the words contagion and infection as contradistinctive terms is to most people very confusing.

Classification of diseases according to this method was always difficult and in the light of our present knowledge seems altogether useless.

Dunglison in his medical dictionary, in defining the terms says: "Contagion and infection are generally considered synonymous. Frequently, however, the former is applied to diseases not produced by contact, as measles, scarlet fever, etc.; while infection is applied to those that require positive contact, as itch, syphilis, etc., and conversely."

The term contagion is understood by most medical men, to mean those diseases which are communicated from one individual to another individual by direct contact. While the term infection refers to the transmission of disease from one individual to another individual by means of some secondary object. That contagion means direct infection and infection means indirect contagion. That is to say a precise line of distinction is attempted to be laid down based upon the mode of communication.

* Read before the United States Veterinary Medical Association.

Then to make matters more confusing it has been attempted by many to subdivide the class of contagious diseases into (1) immediate, (2) mediate; the immediate contagious being the true contagious diseases, *i. e.*, those which are communicated by direct contact; while the mediate are those which are transmitted only through the medium of secondary objects. Thus you will see that the sub-division (2) of class A, contagious diseases, corresponds pretty closely with class B, infectious diseases. The only difference being that the atmosphere was supposed to play some very important part in the propagation of infectious diseases, while that agent was denied to the diseases classified under this method, in subdivision (2) of Class A, the mediate contagious diseases.

Perhaps some such classification as the above may have been permissible when but little was known about the causation of disease, and when clinical evidence was the only basis upon which to work.

Of late, however, with improved instruments at our command and a comparatively perfect technique to aid us, such rapid strides have been made in the study of the ætiology of disease that the maze which formerly clouded the attempted classification of communicable diseases is fast passing away.

It is the opinion of the most recent authorities that the term *infection* means the condition produced by the entrance into the body of pathogenic micro-organisms, and thus multiplication within the body. By the term micro-organism is meant, it must be remembered, not bacteria alone, but all low forms of vegetable or animal life.

If, therefore, all diseases communicable from one individual to another individual are produced by the entrance into the body of pathogenic micro-organisms and their multiplication within the body, it is clear that the words contagious and infectious should not be used as contra-distinctive terms. It is only necessary to come in contact with the micro-organism of an infectious disease to become contaminated if the condition of our tissues is such as to favor the growth and multiplication of that micro-organism. Any disease, therefore, in which the micro-organisms are contained in the fæces is contagious just the same as one in which the infecting element is situated in the skin. Only in the one case the chances of infection are not so great because the probabilities of contact are lessened. We are not, it is to be hoped, in the habit of handling excreta without afterward washing and probably disinfecting our hands. On the other hand we are quite apt to touch the surface of diseased skin without taking any particular precaution against infecting ourselves or other individuals. Then too we must remember that micro-organisms do not rise from moist surfaces, hence the chances of infection is reduced to a minimum in many cases of communicable diseases. It was upon the probable chances of contamination that the basis of the classification of contagious *vs.* infectious diseases was made.

There is, however, a group of diseases which do not answer to the above but which are classified as *miasmatic.* They are quite contradistinctive to the infectious diseases in that while they are due to the introduction of a micro-organism into the body yet those organisms *do not* multiply within the body or at least are discharged in a form suitable of producing infection in another individual. They require a proper nidus outside of the body to complete their development. Hence it is that such a miasmatic disease as malaria is not transmitted directly from one individual to another. Yet the disease is micro-organismal just as much as is anthrax or tuberculosis.

We would recommend, therefore, that the name of this committee be changed by substituting the word miasmatic for the word contagious. In which case it would read "*Committee on Infectious and Miasmatic Diseases.*" Infectious meaning those diseases due to the introduction of pathogenic micro-organisms, which complete their life history within the body, and are therefore capable of directly infecting a second individual. Miasmatic meaning those diseases due to micro-organisms which require a suitable nidus outside of the body to complete their life history. Such a classification is, it seems to us, the only rational one in the light of our present knowledge, and will be much better to the future member of this committee in following the literature of the day for the purpose of obtaining statistical information.

As chairman of this committee, I had printed one hundred circulars asking for information as to the prevalence of certain communicable diseases. I also had printed a list of the diseases upon postal cards, addressed to me, which I enclosed with the circular. These one hundred circulars and postal cards were sent to such persons as I thought would be most likely to have the desired information. The list included the names of representatives of boards of health and State veterinarians in, I think, every State of the Union. Thirty-five people responded to this request.

According to these reports the deaths in the human family from tuberculosis during the past year were in New York City, 5,913. In the State of Rhode Island 750. In the State of New Hampshire about 1,000. In the State of Colorado some 300. Massachusetts reports for 1886, 5,897. The States of California and Maryland reported *no registration*.

The other State boards of health did not respond to the circular.

Of tuberculosis in animals, seventeen veterinarians in active practice reported a total of 303 cases. In the report furnished me by Dr. Geo. C. Faville, Chief of the Bureau of Animal Industry for Maryland, it appears that 169 cows, coming within a radius of six miles from the City Hall in Baltimore, were proven to be tuberculous by post-mortem examination, within the past year. This refers principally to pulmonary tuberculosis, as nearly all of these were dressed for beef, and as it is the business of the inspectors to look out for contagious pleuro-pneumonia almost exclusively, the lungs as a rule are the only organs examined. Were it possible to make a careful post-mortem examination of a large number of the cows comprising the milk dairies in Baltimore or in any of our larger cities, I am certain that the percentage would run very high. It is no uncommon thing to find, in those cows upon which we have an opportunity of making a careful post-mortem examination, *i. e.*, cows dying from tuberculosis or some other form of disease—more or less—extensive tubercula lesions, either not wholly confined to the lungs, or in which the thoracic organs are healthy, while there is either actual or healed tuberculosis in the other organs of the body.

Since Koch discovered the tubercle bacillus it has been proven beyond all doubt that the disease is the same in man and animals. Many forms formerly classified as other than tuberculous are now included under the one head. In fact, wherever tissue changes occur and the presence of the pathogenic bacillus is determined, there we have the disease. While the discovery of the specific bacillus has proven that the disease was much more prevalent than even it had been supposed, it has also aided in demonstrating that tuberculosis can be, and in a very large percentage of cases is completely cured.

I am informed on very good authority that fully fifty per cent. of the post-mortems in our hospitals and in private practice show lesions of tuberculosis. In a large percentage of these cases the lesions are completely healed. Yet, while proper surroundings may do much toward the cure of this disease, the fact remains that one in every seven of the people born die of tuberculosis.

How much the prevalence of tuberculosis among people is due to infection from the lower animals either from the consumption of meat or milk, or from association, is hard to say. Certain it is that the consumption of milk containing the bacillus is to blame for much of the tuberculosis seen in young children. The presence of the bacillus in milk has been demonstrated by Bollinger even when there were no nodules to be found in the udder. We have no less an authority for the statement that in a series of experiments made by him he found that in a lot of cows affected with extensive tuberculosis no less than 80 per cent. of the cases showed infection of the milk. In cows with moderate tuberculosis the milk was infected in 66 per cent., and in cows with slight tuberculosis 33 per cent. of cases.

Dr. Austin Peters, of Boston, Mass., in a letter to me dated August 23, 1889, reports that at the experimental farm at Mattapan, Mass., where in connection with Dr. Ernst he has been making some interesting observations, they have had in all 18 tuberculous cows from ten different herds representing eight towns, all within a radius of twenty-five miles from Boston, except in one instance where a cow came from Newport, R. I. He has killed thirteen calves and seven pigs which had been fed upon the milk of these cows for a period of from three to six months. Two pigs and six calves were tuberculous. Nine of the eighteen cows have been killed and all proved by post-mortem examination to be tuberculous. Tubercle bacilli were found in the milk of six of these cows. He says further, that he has found the bacillus in three other cows that were never in his possession. He has also visited several herds in the State and has found the disease existing in from 1 to 100 per cent of the animals. He also mentions a case which came under his observation where a pet dog became infected by eating the sputum of a tuberculous woman.

It is therefore the opinion of your committee that the individual members of this association should use every opportunity to impress upon the people the danger arising from the consumption of the meat or milk of tuberculous animals. All of our cities should have public abattoirs and there all of the cattle to be used for beef should be killed under the supervision of competent veterinarians. In no other way than by the centralization of the slaughtering can food supply be examined. It is either worth doing well or it is not worth doing at all. To inspire false confidence in the people is a crime. Yet this is done to-day in many of our large cities where men who have no qualifications for the office are employed as so-called meat inspectors, whose duty it is to walk about and go into a shop here and there, examine a piece of meat to see whether it smells bad or not, not knowing that the most foul smelling organisms are as a rule the least dangerous.

Our milk dairies should be inspected often, and where there is the least suspicion the milk should be subjected to a microscopic examination to determine the presence or absence of the tubercular bacillus. This should, obviously, be done at the expense of the community supplied, as they are the ones most interested. Whenever it is found necessary to condemn an animal that animal should be paid for out of the common fund unless it can be proved that the owner put it there with malicious intent, when the law should make him suffer severely.

*Glanders.*—Dr. Edson, of the Health Department for New York City, reports 1 case of glanders in man and 125 in animals.

Dr. Paul Paquin, State Veterinarian for Missouri, reports 5 cases of glanders in man and 137 cases in animals in the state. These are the only cases reported in man, but from the meager opportunities which physicians have of studying the disease, as compared with veterinarians, it is quite possible that deaths occur from glanders in human beings without the disease being diagnosed. I have known, personally, of one case where but from the fact that the physician who was called in consultation had seen one or two similar cases, the patient would have died before a correct diagnosis had been arrived at.

From the reports furnished me by veterinarians, it would seem that the disease in horses was pretty evenly distributed throughout the United States. From the fact that it entails a great commercial loss and as the possibilities of human infection, the disease should, if possible, be exterminated. At any rate the laws should be strict enough and their enforcement should be thorough enough to prevent the offering for sale, or driving or leading upon the streets animals known to have glanders. The positive diagnosis of the disease is in many cases impossible and often difficult, so that the veterinarian should use great care in rendering a decision. If there are reasonable grounds for suspicion, however, even though the evidence be not very conclusive, the attending surgeon is justifiable in quarantining the animals. A guinea-pig should be inoculated with any discharge that may appear, or, as has lately been recommended, the sub-maxillary lymphatic glands may be excised and an animal inoculated for diagnostic purposes.

*Actinomycosis.*—One case in a human being was reported lately by Dr. Large in New York. This disease is quite prevalent in cattle throughout the country. It may attack almost any part of the body, though as you all know, it most commonly occurs in the bones of the face. I have seen cases where in addition to the affection of the jaw, the tumors were found posterior to the pharynx and in the lung. In several instances I have seen it in the lungs where there were no tumors present in any other part of the body. Several months since I saw at one of the slaughter-houses in Baltimore a tumor about the size of a man's fist growing from the anterior border of the sheath of a stud's penis. There were no tumors to be found in any other part of the body. Microscopical examination proved this to be actinomycosis.

I do not know how prevalent the disease is in hogs in this country, but I have seen it often enough in Germany, infesting the muscle as minute whitish dots scarcely larger than a pin's point, and in many cases only visible on microscopic examination. The infection when it occurs in hogs seems to be much more general than it is in cattle, and much more difficult of recognition; there are seldom or never any large tumors.

*Trichinosis.*—One case in man is reported from New York. I have not been able to obtain any statistics as to its prevalence in hogs except those published some few years ago. Dr. F. S. Billings sets the number of hogs infected, I believe, as 1 in 17. These hogs came from the Western States. Of 1000 hogs examined by myself in Canada in 1883, but four had trichinosis. Why it should be so much more prevalent in the Western States than in Canada I am unable to say.

*Cestode Tuberculosis.*—Dr. Paquin reports 36 cases in cattle. None are reported in pigs.

Cestode tuberculosis in cattle is caused, as you know, by the encysted larval form of the Taenia Medio-canellata, the adult beef tape worm found in man.

Cestode tuberculosis in pigs is the encysted larval form of the Taenia solium, or adult pork tape worm found in the intestine of man.

The encysted form in cattle is very small and hard to find. The encysted form in pigs is much larger and hence easier of recognition. Yet the cysts must be fully as common in cattle as in pigs, because the Taenia Medio-canellata is much more prevalent in man than is the Taenia solium. Perhaps the fact that pork is much more thoroughly cooked has much to do with lessening the chances of infection with the pork as compared with the beef tape worm. Although the presence of these worms in man's intestines is not dangerous to life, nevertheless they are a great source of discomfort, and could, in many cases, be prevented by a proper meat inspection. In Montreal, Canada, in 1883, out of a population of 180,000 there were 200 cases of people affected with tape worms. Care should be taken in the examination of animals not to mistake the larval forms of the T. Medio-canellata and the T. solium, the adult tape worm of man, for the larvæ of the Taenia echinococcus, the adult tape worm of the dog. The larvæ of the two former are softer, the membrane forming the cyst wall is more translucent, and through it can be seen a minute opaque white body, the head and neck of the immature worm. Taenia echinococcus cysts on the other hand are harder, and of an opaque yellow color. When ruptured, a clear fluid is, as a rule, discharged, the secondary buds rarely being present in animals as a rule, the individuals harboring them are not allowed to live long enough for the full development of the cysts. No harm can come to man by the consumption of the larvæ of T. echinococcus. On the other hand, if a dog were to eat them the Taenia echinococcus in adult form would be the result, as the dog's intestine is the natural habit for this worm. The danger to man comes from getting, in some way, the eggs of the mature worm into his body, where cysts may form the same as in cattle or pigs. Echinococcus disease is very rare in this country, but in Iceland, where association between dog and master is very intimate, and where the habits of the people are not as they might be, the disease is much more prevalent.

*Contagious Pleuro-pneumonia.*—This disease certainly seems to be on the decrease. In Maryland, during the past year, according to the Bureau of Animal Industry reports, 107 head of cattle have been proven by post-mortem examination to have had contagious pleuro-pneumonia. For some time past, however, no undoubted cases have been found, and it is not known at present that there are any herds in Maryland infected. Unfortunately we have not been able to get at the æteology of the disease. It is to be hoped that some one will yet be fortunate enough to get at the bottom of the trouble.

*Infectious Pneumonia.*—By this term is meant that form of pneumonia in horses generally spoken of as the influenza type. From reports received the disease is quite prevalent and pretty evenly distributed throughout the country. Schütz, of Berlin, has pretty well demonstrated the infectious nature of the disease, but time will not allow me to go into details to-day.

*Hog Cholera.*—This disease is pretty widely distributed, and although it is held by many to be closely allied to another disease which Dr. Salmon has named Swine Plague, yet they have not been separated clinically, and were we to go into the details of the arguments for and against the recognition of two separate diseases, the report of your committee would have to be confined to this one subject. In an able paper read before this association, Dr. Salmon defended his side of the question, and very likely other papers upon this malady may be forthcoming.

*Chicken Cholera* seems to be prevalent just now. Your committee has nothing now to offer on the subject, however.

*Texas Fever*, as is usual at this time of the year, is reported as prevalent. Either this disease, or one very similar, is particularly prevalent among milk cows in Baltimore, and without any history of contact with Texas cattle.

*Rabies.*—Dr. C. C. Lyford, of St. Paul, reports three cases in men, one in a dog, and one in a cat. Twelve cases of the disease were reported by different veterinarians.

In conclusion, gentlemen, allow me to thank those who responded so promptly and to whom I am indebted for much valuable information. It is to be hoped that a greater number of circulars will be sent out by the next chairman, and that all will feel an interest in the subject by sending to this committee such information as they may have in their possession.

